# Early and late onset sepsis and retinopathy of prematurity in a cohort of preterm infants

**DOI:** 10.1038/s41598-022-15804-4

**Published:** 2022-07-08

**Authors:** Elena Bonafiglia, Elena Gusson, Rosa Longo, Benjamim Ficial, Maria Giulia Tisato, Sara Rossignoli, Giulia Caltran, Emilio Pedrotti, Renzo Beghini, Giorgio Marchini

**Affiliations:** 1 Neonatal Intensive Care Unit, Mother and Child Department, University Hospital of Verona, P. le A. Stefani 1, 37126 Verona, Italy; 2 Ophthalmic Unit, Department of Neurosciences, Biomedicine and Movement Sciences, University of Verona, Verona, Italy; 3 Department of Diagnostics and Public Health, University of Verona, Verona, Italy

**Keywords:** Paediatric research, Eye diseases, Retinal diseases, Retinopathy of prematurity, Neonatal sepsis

## Abstract

This study investigates the impact of antenatal and postnatal infection or inflammation on the onset and progression of Retinopathy of Prematurity (ROP). We retrospectively collected clinical and demographic data of preterm infants with birth weight ≤ 1500 g or gestational age < 30 weeks admitted to the neonatal intensive care unit of Verona from 2015 to 2019. Uni- and multivariable analysis was performed to evaluate the potential effect of selected variables on the occurrence of any stage ROP and its progression to severe ROP, defined as ROP requiring treatment. Two hundred and eighty neonates were enrolled and 60 of them developed ROP (21.4%). Oxygen need for 28 days and late-onset sepsis (LOS) increased the risk of any grade ROP after adjusting for birth weight and gestational age (OR 6.35, 95% CI 2.14–18.85 and OR 2.49, 95% CI 1.04–5.94, respectively). Days of mechanical ventilation and of non-invasive ventilation increased the risk of progression to severe ROP after adjusting for birth weight and gestational age (OR 1.08, CI 1.02–1.14 and OR 1.06, CI 1.01–1.11, respectively). Exposure to infection with production of inflammatory mediators may contribute to increase the risk of ROP occurrence in very preterm neonates.

## Introduction

Retinopathy of prematurity (ROP) is a vascular proliferative disease that affects preterm infants. Following the increased survival of more preterm neonates, due to advances in neonatal care, ROP continues to be one of the primary causes of potentially treatable lifelong visual impairment and blindness in childhood, in both high and middle-income countries^1^.

Routine screening program based on birth weight and gestational age allows timely diagnosis of neonates and prompt treatment in those with sight-threatening ROP, thus limiting ROP-related morbidity^2^.

Recently, predictive algorithms that take into account postnatal risk factors have been proposed to individualize risk prediction and allow better risk stratification. These algorithms early identify infants that will eventually develop a severe form of ROP, far in advance compared to appearance of retinal lesions. Although further validation is needed before incorporation in clinical practice, predictive algorithms have the potential to reduce both the number of neonates undergoing screening and the number of missed or late diagnosis^3,4^.

ROP pathogenesis is divided into two sequential phases. In the first phase, the immature retina is exposed to higher levels of oxygen (relative hyperoxia). The latter causes an arrest of retinal vascularization. In the second phase, the undeveloped retinal blood vessels are unable to provide an adequate oxygen supply for the increased metabolic demand of the more mature retina, leading to an upregulation of retinal growth factors (including VEGF) and an aberrant neo-vascularization. This may ultimately lead to retinal detachment and permanent vision loss^5^.

Although risk factors for ROP, such as low gestational age, low birth weight and oxygen requirements, are well known, the precise mechanisms leading to occurrence of ROP have not been fully understood^2^. Recent studies suggest a potential role of both prenatal and postnatal infection, however both the occurrence of ROP and its progression from the first to the second phase has not been completely clarified^6^. In fact, infection could trigger the production of inflammatory markers (cytokines, chemokines, growth factors, etc.) that contribute to both arrest of vascularization in the first phase of ROP and aberrant neo-vascularization in the second phase^7^.

Though these initial findings are promising, a better understanding of the role of infection in the ROP pathogenesis is needed. The latter would eventually allow to better individualize risk prediction on the basis of prenatal and postnatal infections and to identify potentially preventable risk factors. Finally, identification of the specific molecular pathways involved in ROP pathogenesis may help the future development of new treatments.

Aim of this study was to investigate the impact of antenatal and postnatal infections on the onset and progression of ROP in a cohort of very preterm neonates.

## Materials and methods

This was an observational retrospective study carried out at the level three Neonatal Intensive Care Unit (NICU) of Verona (Italy), from January 2015 to December 2019. Ethics approval was obtained from our local research ethics committee (“Comitato etico per la Sperimentazione Clinica delle Province di Verona e Rovigo”). The parents or guardians of babies enrolled gave their written informed consent. All procedures performed in studies involving human participants were in accordance with the ethical standards of the institutional and/or national research committee and with the 1964 Helsinki declaration and its later amendments or comparable ethical standards. All infants admitted within the study period who met the eligibility criteria were enrolled.

No formal sample size calculation was done. Inclusion criteria were the ROP screening criteria according to the American Academy of Pediatrics recommendation: birth weight ≤ 1500 g or gestational age ≤ 30 weeks^8^. Exclusion criteria were: death or transfer before ROP screening, major congenital malformations, chromosomal disorders, inherited metabolic diseases.

ROP screening was performed by two board certified consultant ophthalmologists (EG, RL), according to the recommendation of the American Academy of Pediatrics (AAP)^8^. Retinal screening examinations were performed after pupillary dilation with 2.5% phenylephrine galenic eye drops by using binocular indirect ophthalmoscopy. The international classification of retinopathy of prematurity revisited (ICROP3) was used for disease staging^9^. Timing of first eye examination was based on gestational age at birth and follow-up examinations were based on retinal findings, following the AAP recommendation. All patients were followed up until completion of vascularization, even after discharge from the NICU. Infants with stage 1 ROP or incomplete vascularization were evaluated every 2 weeks. Patients with stage 2 or higher were evaluated weekly. In the presence of plus sign, the newborns were evaluated several times a week. The RetCam Imaging System III (Clarity Medical Systems, Pleasanton, CA) was used in any grade ROP to confirm diagnosis, monitor disease and treatment, evaluate retinal neovascularization and to take a fundus photo.

All diagnosed ROPs were included as the disease progresses in stages, except for aggressive ROP, which does not follow the classic pattern of progression. It was further distinguished between severe ROP (or Type 1 ROP) and Type 2 ROP. The former includes ROP in zone I of any stage with plus sign, stage 3 with and without plus sign and zone II stage 2 or 3 with plus sign. The second includes ROP zone I stage 1 or 2 without plus and zone II stage 3 without plus. The above-mentioned distinction is pivotal, as it allows to distinguish between Type 1 ROP or severe ROP, that requires treatment, and Type 2 ROP, that requires only monitoring, until completion of the vascularization. This distinction is also one of gravity, as Type 2 ROP typically has minor consequences^10^.

Clinical and demographic data were collected from electronic medical records. The following data were collected: antenatal steroid use, antenatal antibiotics administration, intrauterine growth restriction (IUGR), preterm premature rupture of membranes (PPROM), clinical and histological chorioamnionitis, maternal C-reactive protein (CRP), maternal rectal and vaginal swab, gestational age at birth, birth weight, length and head circumference, sex, Apgar score at 5 min, mode of delivery, single or multiple birth, admission blood culture, surface (ear, nose, perineal) swabs at admission, number of surfactant doses, duration of non-invasive respiratory support and mechanical ventilation (days); duration of parenteral nutrition (days), number of red blood cell (RBC), fresh frozen plasma (FFP) and platelet (PLT) transfusions, occurrence of early-onset sepsis (EOS), late-onset sepsis (LOS), patent ductus arteriosus (PDA) requiring medical and/or surgical treatment, bronchopulmonary dysplasia (BPD), defined by oxygen need for 28 days and an assessment of respiratory support at 36 weeks’ postmenstrual age^11^, necrotizing enterocolitis (NEC), according to Bell’s diagnostic criteria and need of surgical intervention^12^, intraventricular hemorrhage (IVH), according to Papile’s classification^13^, death before discharge. For each infant, we reported the presence of ROP, stage and treatment, if any.

Clinical chorioamnionitis was defined according to the conventional Gibbs criteria: the presence of fever (≥ 37.8 °C) and more than two of the following: maternal leucocytosis (white blood cell count > 15,000 cell/mm^3^), maternal tachycardia (heart rate > 100 beats/min), fetal tachycardia (heart rate > 160 beats/min), uterine tenderness and foul-smelling vaginal discharge^14^. EOS and LOS were defined when a bacterial pathogen was isolated from a blood and/or cerebrospinal fluid culture before and after the first three days of life, respectively^15^. If a Coagulase negative staphylococcus (CONS) was detected, sepsis diagnosis was confirmed if there was CONS growth in two consecutive blood cultures taken within two days or with CONS growth in a single blood culture along with CRP elevation^16^.

Relevant treatment protocols did not change during the study period: surfactant was given as early rescue therapy, target SpO2 was from 90 to 95%, aim of respiratory support was to maintain pH > 7.20, and SpO2 from 90 to 95%, transfusion protocol followed the recommendations of the Italian society of neonatology.

### Statistical analysis

The neonates enrolled in the study were divided into two subgroups: neonates who developed any stage ROP or those who did not. Normality of data distribution was assessed by Shapiro–Wilk’s test. Data were presented as mean (SD), median (interquartile range (IQR)) or count (%) for parametric, non-parametric and categorical variables respectively. To compare the two groups of neonates independent-sample t-test, Mann–Whitney U test and χ^2^ test were used in case of parametric, non-parametric and categorical variables respectively.

Potential independent effect variables on the occurrence of any grade of ROP and of ROP progression to severe ROP or Type 1 ROP were selected by univariable analyses. Variables that were highly correlated with others (variance inflation factors or VIF above 5) and those with no predictive value (P value > 0.10) were removed from the final model. A multivariable stepwise logistic regression analysis was performed to evaluate the potential independent effect of the selected variables on the occurrence of any stage ROP and of severe ROP. Effect estimates were expressed as odds ratio (OR) and 95% confidence interval (95% CI). Data were analysed with IBM SPSS 23 (SPSS, Chicago, Illinois, USA). P value < 0.05 was considered significant.

## Results

**Figure 1:**
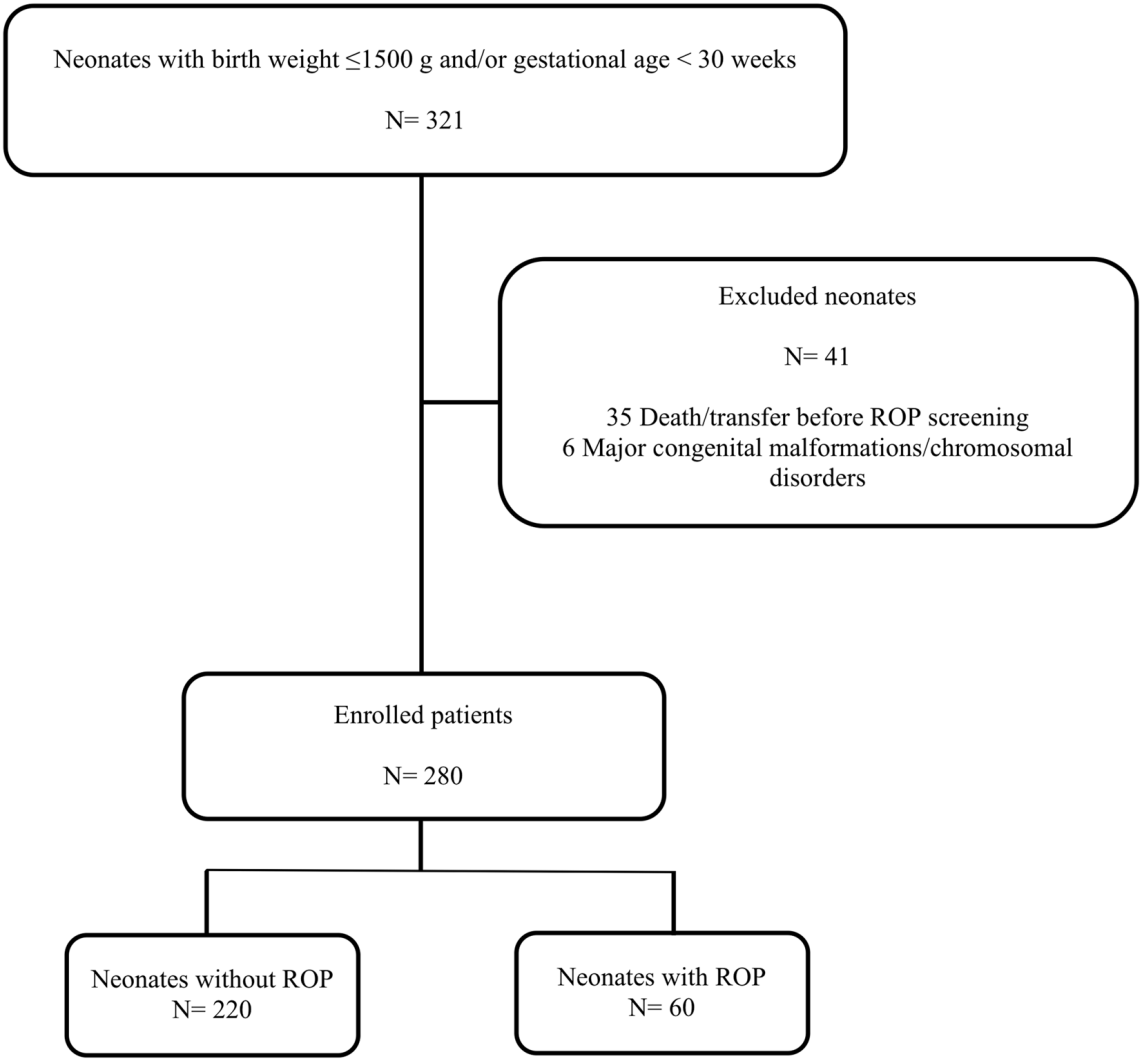
Study flow diagram. ROP, Retinopathy of prematurity.

During the study period 321 neonates with birth weight ≤ 1500 g and/or gestational age ≤ 30 weeks were admitted to our NICU. Forty-one neonates were excluded due to major congenital malformations/chromosomal abnormalities (n = 6) and death or transfer before ROP screening (n = 35). A total of 280 neonates were studied (Fig. 1).

Sixty neonates developed ROP (21.4%). Stage of ROP at diagnosis and at discharge according to ICROP-3 is reported in Table 1. Among patients with stage 1 ROP (n = 31), 3 were discharged at stage 1, the others recovered completely. All stage 2 neonates (n = 11) had complete regression of disease, except one who was transferred to another hospital with incomplete regression to stage 1.

**Table 1:** ROP stages at diagnosis and discharge of included neonates. Values are presented as count (%).

Stage of ROP	At diagnosis (n = 60)	At discharge (n = 60)
Stage 0	–	44 (73.3)
Stage 1	31 (51.7)	4 (6.7)
Stage 2	11 (18.3)	0 (0)
Stage 3	15 (25.0)	0 (0)
Stage 4	3 (5.0)	0 (0)
Stage 5	0 (0)	0 (0)
Treatment outcomes	–	12 (20)

Among neonates with stage 3 ROP (n = 15), 6 regressed to lower stages until healing and completion of vascularization, 9 were treated with laser photocoagulation after the onset of plus sign. Infants with stage 4 ROP (n = 3) were treated with pars plana vitrectomy with endo-laser photocoagulation.

Clinical characteristics of the two cohorts of neonates with and without ROP are presented in Table 2. Neonates who developed ROP had lower gestational age, birth weight, length and head circumference at birth, longer need of mechanical ventilation and non-invasive ventilation, more frequent need of surfactant doses, RBC, PLT and FFP transfusion, more frequent complications such as IVH, oxygen need for 28 days, LOS, PDA, NEC, maternal chorioamnionitis.

**Table 2:** Clinical characteristics of the two cohorts of neonates with and without ROP. Values are presented as median [IQR], and count (%).

	ROP (60 neonates)	No ROP (220 neonates)	p
Female	31 (51.7%)	111(50.5%)	0.86
Gestation (week)	26.2 [25.1–28.5]	29.8 [28.6–31.3]	< 0.05
Birth weight (g)	857 [735–1007]	1285 [1056–1400]	< 0.05
Lenght at birth (cm)	33 [31–36]	38 [36–40]	< 0.05
Head circumference at birth (cm)	24 [22.5–25.8]	27.5 [26–28.5]	< 0.05
IUGR	9 (15%)	54 (24.5%)	0.11
Positive maternal vaginal swab	17 (43.6%)	41 (35%)	0.33
Positive maternal rectal swab	12 (30.8%)	36 (31.3%)	0.95
PPROM > 18 h	24 (40%)	63 (28.8%)	0.19
Maternal fever during labor	8 (14%)	21 (9.8%)	0.35
High maternal CRP	25 (48.1%)	63 (38.4%)	0.21
Histological chorioamnionitis	23 (39.7%)	57 (27%)	0.06
Clinical chorioamnionitis	16 (27.6%)	24 (15.8%)	< 0.05
Cesarean delivery	39 (65%)	182 (82.7%)	< 0.05
Multiple birth	15 (25%)	81 (36.8%)	0.10
Antenatal antiniotics	38 (63.3%)	143 (65%)	0.81
Antenatal steroids (partial or complete)	56 (93.3%)	215 (97.7%)	0.09
5-Min Apgar score	7 [6–9]	8 [8–9]	< 0.05
Positive blood culture at birth	1 (1.7%)	5 (2.3%)	0.78
Positive swab at birth	4 (6.8%)	15 (7%)	0.95
Surfactant doses (n)	1 [1–2]	0 [0–1]	< 0.05
Mechanical ventilation duration (days)	13 [4–37]	0 [0–2]	< 0.05
Non-invasive respiratory support duration (days)	42 [32–60]	14 [5–27]	< 0.05
Oxygen need for 28 days	55 (91.7%)	65 (29.5%)	< 0.05
Medical treatment of PDA	42 (70%)	34 (15.5%)	< 0.05
PDA ligation	6 (10%)	2 (0.9%)	< 0.05
Grade of IVH	0 [0–2]	0 [0–0]	< 0.05
NEC requiring surgery	10 (16.7%)	6 (2.7%)	< 0.05
Parenteral nutrition duration (days)	27 [17–45]	12 [7–17]	< 0.05
Early onset sepsis	0 (0%)	7 (3.2%)	0.16
Late onset sepsis	50 (83.3%)	85 (38.6%)	< 0.05
Platelet transfusion (n)	0 [0–1]	0 [0–0]	< 0.05
GRC transfusion (n)	2 [1–2]	0 [0–1]	< 0.05
FFP transfusion (n)	0 [0–1]	0 [0–0]	< 0.05
Death before discharge	1 (1.7%)	0 (0%)	0.05

Univariable analysis identified the following potential predictors of any grade ROP: gestational age, birth weight, oxygen need for 28 days, PDA, late onset sepsis, clinical chorioamnionitis, PLT, FFP and RBC transfusions. Four variables were included in the final model: birth weight, gestational age, LOS and oxygen need for 28 days. The other variables were excluded due to collinearity (VIF > 5). The logistic regression model was statistically significant, χ^2^(4) = 112.948, p < 0.05. The area under the ROC curve (AUC) showed an excellent level of discrimination (AUC 0.901, 95% CI 0.861–0.941). Predictor variables are reported in Table 3. Regression analysis demonstrated that both oxygen need for 28 days and LOS increased the risk of developing ROP (OR 6.35, 95% CI 2.14–18.85 and OR 2.49, 95% CI 1.04–5.94, respectively), after adjusting for birth weight and gestational age. Results of collinearity diagnostics among variables included in the regression model are reported in Table 4 (supplemental file).

**Table 3:** Results from the logistic regression analysis predicting likelihood of any grade ROP.

Predictor variable	Unstandardized ß	Odd ratio	95% CI	p
Birth weight	− 0.003	0.99	0.99–0.99	0.013
Oxygen need for 28 days	1.849	6.35	2.14–18.85	0.001
Gestational age	− 0.182	0.83	0.66–1.05	0.124
LOS	0.915	2.49	1.04–5.94	0.039

When evaluating the predictor variables of severe ROP among neonates with any grade ROP, we found that days of mechanical ventilation and of non-invasive ventilation increased the risk of severe ROP (OR 1.08, CI 1.02–1.14 and OR 1.06, CI 1.01–1.11, respectively), after adjusting for birth weight and gestational age. Clinical chorioamnionitis was not associated with increased risk of severe ROP (OR 4.02 95%, CI 0.97–16.60).

## Discussion

In the current study, we evaluated the association between ROP and maternal and neonatal infection or inflammation in a cohort of very preterm neonates.

In our cohort of very preterm neonates ROP prevalence was 21.4%, similar to what has previously been reported^13,17^.

In our population we found that LOS increased more than twice the risk of developing any stage ROP. The latter is in agreement with previous studies. Reyes et al. in a retrospective cohort of 171 neonates and Chen et al. in a case–control study of 622 neonates found an OR between late-onset sepsis and any stage ROP of 7.47 (95% CI 1.21–46.04) and of 2.1 (1.4–3.2), respectively^18,19^. On the contrary, Cantey et al. in a retrospective cohort of 2242 neonates found that LOS caused only by CoNS and not by all pathogens was not associated with increased risk of any stage ROP (OR 0.86, 95% CI 0.54–1.37, p > 0.05)^20^.

In a recent meta-analysis by Huang et al. aiming to evaluate the association between sepsis and ROP, the pooled OR of the association between any stage ROP and LOS was 1.81 (95% CI 0.74–4.43); however, among 34 included studies, only three discriminated between EOS and LOS^21^. In conclusion, our finding that LOS increased the risk of ROP development supports prior evidence, although the underlying physiological mechanisms are yet to be clarified. We speculate that inflammatory factors triggered by postnatal sepsis, such as growth factors and cytokines (IL-1, TNF-α, etc.), may modulate retinal angiogenesis and alter vessels development^22^.

However, we did not find any association between any grade ROP and EOS or other risk factors of intrauterine infection (maternal chorioamnionitis), as previously reported^23,24^.

It has been proposed that prenatal inflammatory exposure has a limited impact on ROP development, because it occurs in an early phase of retinal development, prior to the first phase of ROP pathogenesis^25^.

Our data seem to support this theory. A recent meta-analysis by Villamor-Martinez et al. found that chorioamnionitis did not increase the risk of severe ROP, after controlling for gestational age. Part of the effects of chorioamnionitis on the development of ROP may be mediated by its role as an etiological factor for very preterm birth^26^. However, there may be an association between prenatal infection or inflammation and ROP that was not captured by the risk factors we examined.

In conclusion, multiple exposures to infection or inflammation in postnatal life may contribute to gradually increase the risk of ROP development (multi-hit hypothesis)^2,27^.

The important impact of LOS on ROP development underlines the importance of developing strategies to prevent nosocomial infections in very preterm babies during their NICU stay. Moreover, we feel that, if an episode of LOS occurs, neonates should be screened more frequently, due to the increased risk of ROP development, although more prospective data are needed before this may be universally recommended in clinical practice.

In the near future, individualizing risk prediction may allow better risk stratification and more sustainable screening program. According to our findings, LOS, that so far has not been included in any algorithm to predict ROP risk, should be further investigated. Larger datasets and further validation are needed before incorporating LOS in ROP score predictive algorithms.

The main limitation of the current study is related to its observational design: although we confirmed an association between LOS and ROP, causality should be still considered uncertain. Moreover, sample size was relatively small. On the other hand, our cohort was homogenous. In fact, during the study period, institutional protocols of stabilization and management of VLBW infants, ventilation and oxygen target saturations did not change.

Another limitation was the lack of severe ROP cases, that reduces the reliability of ROP progression prediction. Further data are needed to confirm the role of infection or inflammation in ROP progression and the multi-hit hypothesis.

Finally, in the current study ROP assessment was based only on disease stage without considering presence and location of the plus disease and zone, to indicate the degree of retinal vessel immaturity. In a future study, we would like to explore if infection or inflammation leads to different subtypes of severe ROP in a larger cohort of patients.

## Supplementary Information

Supplementary Information.

## Data Availability

Data are available upon reasonable request.
