# Supplementary material for: Early and late onset sepsis and retinopathy of prematurity in a cohort of preterm infants
Source: Sci Rep. 2022 Jul 8;12:11675. doi: 10.1038/s41598-022-15804-4 (PMC9270376; doi:10.1038/s41598-022-15804-4)
Supplement: Supplementary file 1 — Supplementary Information. [file 41598_2022_15804_MOESM1_ESM.docx]

**Table 4.**

**Results of collinearity diagnostics among variables included in the regression model on the occurrence of any grade of ROP.**

|  | **Tolerance** | **VIF** |
| --- | --- | --- |
| Gestation (week) | 0.42 | 2.34 |
| Birth weight (g) | 0.49 | 2.02 |
| BPD | 0.53 | 1.87 |
| Late onset sepsis | 0.76 | 1.30 |

*VIF= variance inflation factors*
